# Bioavailability of rumen-protected L-methionine supplement in lactating dairy cows

**DOI:** 10.3168/jdsc.2026-1016

**Published:** 2026-06-25

**Authors:** Rafael A. Menezes, Nancy L. Whitehouse, Dani O'Bryan

**Affiliations:** 1 Department of Agriculture, Nutrition, and Food Systems, University of New Hampshire, Durham, NH 03824; 2 Berg+Schmidt Animal Nutrition, Aurora, IL 60502

## Abstract

Rumen-protected AA are largely used to supply the MP in dairy diets. Rumen-protected methionine (RP-Met) supplements differ in their proportion of the biologically active L-form isomer and in their bioavailability. Based on the plasma free AA dose-response approach, we determined the bioavailability of L-Met60 (LipoAktiv L-methionine 60), a rumen-protected L-Met supplement, and evaluated its effects on plasma AA profile. Ten multiparous Holstein cows in mid lactation were used in a replicated 5 × 5 Latin square design with 7-d experimental periods. All treatments used L-Met60, with doses and routes of administration as follows: 0 (control), 18 (18IL), or 36 (36IL) g/d supplied by continuous abomasal infusion, and 18 (18FL) or 36 (36FL) g/d as a fed supplement mixed in the TMR. Abomasal infusion was delivered continuously and uniformly into the abomasum via rumen cannula using a peristaltic pump, whereas fed L-Met60 was consumed before each meal. Milk and blood samples were collected during the last 3 d of the covariate and the experimental periods. No treatment effects were observed for animal performance, except for a discrete increase of 0.06 percentage units in milk protein for 36IL. Infused and fed L-Met60 supplementation did not affect plasma metabolites, but altered plasma Met, cystine, cystathionine and allocystathionine, glutamine, taurine, and total sulfur AA (TSAA). These changes, especially for taurine and glutamine, might be explained by a higher activation of the transsulfuration pathway. Plasma TSAA increased linearly with both routes of administration; however, the regression analysis showed steeper slope for abomasal infusion than for the fed supplementation. Based on this response, the relative bioavailability of L-Met60 was calculated as 43.4 ± 2.5% (TSAA, % of [total AA − TSAA]).

## Highlights

• Plasma Met, TSAA, and cystine increased only with abomasal infusion.
• Infused L-Met at 36 and 18 grams/day, as well as fed L-Met at 36 grams/day, increased plasma taurine.
• Plasma glutamine was decreased, likely due to transsulfuration pathway stimulation.
• The relative bioavailability of fed L-Met60 was 43.4% ± 2.5%.

## Graphical Abstract

**Figure:**
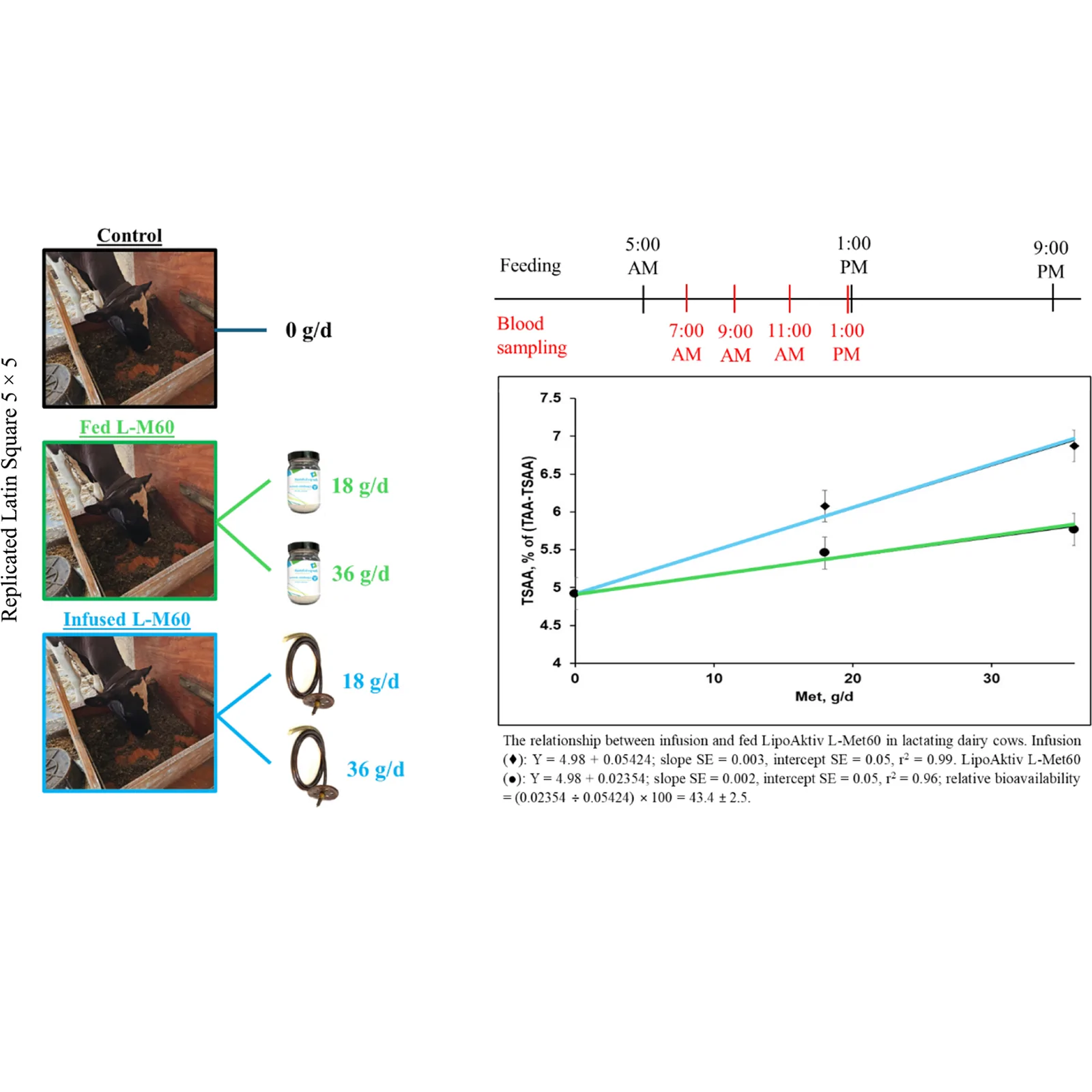
**Summary:** Knowing the bioavailability of rumen-protected methionine (Met) supplements is essential to optimize their inclusion in diets for high-producing dairy cows. The objective of this study was to determine the bioavailability of a rumen-protected Met supplement (L-Met60) using the plasma amino acid dose-response technique. No major effects were observed on animal performance or plasma metabolites. In contrast, concentrations of some plasma amino acids were influenced by L-Met60 supplementation. Moreover, abomasal infusion of L-Met60 resulted in higher plasma total sulfur amino acid (TSAA) concentrations and a steeper slope in the regression analysis compared with fed supplement. The relative bioavailability of L-Met60 was estimated at 43.4% (TSAA % of [TAA - TSAA]). TAA = total amino acids.

The bioavailability of rumen-protected AA, such as lysine and methionine (Met), has been evaluated using the plasma free AA dose-response technique to support their efficient use in dairy diets (Rulquin and Kowalczyk, 2003; Whitehouse et al., 2017). This approach allows the estimation of metabolizable AA supply from protected products under in vivo conditions and has been widely used to compare different sources of rumen-protected AA (Rulquin and Kowalczyk, 2003; Whitehouse et al., 2017; Fagundes et al., 2022). Different methionine supplements vary according to their D:L isomer ratio and physicochemical characteristics, including the encapsulation matrix, which influence their protection against ruminal degradation and their subsequent digestion in the gastrointestinal tract. As a result, the bioavailability of rumen-protected methionine (**RP-Met**) products may vary considerably among commercial sources. Therefore, evaluating the bioavailability of specific RP-Met products is demanded for both ruminant research and industry to determine appropriate supplementation rates for balanced diet formulation. Accordingly, the objective of this study was to determine the relative bioavailability of a rumen-protected L-methionine supplement (**L-Met60**) using the plasma free AA dose-response technique in lactating dairy cows. We hypothesized that supplementation with rumen-protected L-methionine would increase metabolizable methionine supply and result in dose-dependent changes in circulating sulfur AA.

All procedures were approved by the University of New Hampshire Institutional Animal Care and Use Committee (protocol no. 220803) and conducted at the University of New Hampshire Fairchild Dairy Teaching and Research Center (Durham, NH).

Ten multiparous Holstein cows in mid lactation (122 ± 25 DIM; 734 ± 72 kg BW; 24.2 ± 2.7 kg/d DMI; 40.9 ± 4.8 kg/d milk yield; ± SD) were used in a replicated 5 × 5 Latin square design with 7-d experimental periods. Each period consisted of a 4-d adaptation phase followed by a 3-d sampling phase (Whitehouse et al., 2024). Baseline measurements were collected once before the initiation of experimental treatments and were included in the statistical analysis as covariates to account for initial variation among cows. All treatments used **L-Met60** (LipoAktiv L-Met60; Berg+Schmidt Animal Nutrition, Hamburg, Germany), a rumen-protected methionine supplement containing 60% L-methionine. The amount of L-Met60 to be fed or infused was determined using the NRC (2001). The basal diet was entered into the NRC model, and L-Met60 was incrementally added until the predicted Met requirement was met. This corresponded to 16 g/d of L-Met60, assuming a relative bioavailability of 50%

Treatments consisted of different doses and routes of administration: 0 g/d (control), 18 g/d (**18IL**), or 36 g/d (**36IL**) supplied by continuous abomasal infusion, and 18 g/d (**18FL**) or 36 g/d (**36FL**) supplied as a fed supplement, previously mixed with 1.0 kg of TMR, placed in rubber buckets, and then offered individually to the animals 30 min before each feeding. Any residual mixture was placed directly into the rumen via ruminal cannula to ensure complete consumption of the supplement. Immediately after each feeding, the supplement was mixed with the TMR and stored at 4°C (39°F) until the subsequent feeding.

Abomasal infusion was delivered continuously into the abomasum via rumen cannula using a peristaltic pump (Masterflex, Cole-Parmer, Vernon Hills, IL). Fresh infusion solutions were prepared daily by dissolving L-Met60 in approximately 4 L of hot water to ensure complete dissolution. The pump was inspected daily, 5 times per day (0530, 0900, 1300, 1700, and 2100 h) to confirm proper operation and to make adjustments if necessary.

The basal diet was formulated using the Cornell Net Carbohydrate and Protein System (CNCPS) model implemented in Agricultural Modeling and Training Systems (AMTS; version 4.18.6) as TMR based on expected DMI and milk production of the experimental cows. The ingredient composition included (on a DM basis) 34.3% mature corn silage, 15.9% mid-maturity mixed mostly grass silage, 4.11% steam-flaked corn, 12.7% corn meal, 9.72% beet pulp, 0.90% distillers grains with solubles, 8.61% soybean meal, 2.67% canola meal, 4.74% AminoMax (a heat-treated soybean canola, Afgritech, LLC Watertown, NY), 1.13% molasses, 0.12% urea, 2.74% rumen-protected fat (BergaFat F-100; Berg+Schmidt Animal Nutrition, Hamburg, Germany), and 2.40% mineral–vitamin mix. Chemical composition of the basal diet (TMR) is presented in Table 1. Cows had free access to water and were fed individually 3 times daily (0500, 1300, and 2100 h) with approximately one-third of their daily feed allotment at each feeding.

**Table 1:** Ingredient composition of the basal diet fed to multiparous lactating Holstein cows receiving 16 or 32 g/d of rumen-protected methionine supplementation via TMR or infusion

Nutrient composition^1^	DM %
CP	15.3
NDF	29.1
ADF	19.8
Starch	25.2
Sugars	4.26
Ether extract	6.10
Ash	5.34
NEL, Mcal/kg	1.68
NEL required, Mcal/d	42.6
NEL supplied, Mcal/d	40.6
NEL balance, Mcal/d	−2.0

1 Values were calculated from the ingredient composition of the diet using the analyzed chemical composition of individual ingredients on a DM basis. Ingredient analysis done by wet chemistry (Dairy One, Ithaca, NY).

Milk and blood samples were collected during the last 3 d of both the covariate and treatment phases of each experimental period. Blood samples were collected from the coccygeal vein 4 times per day at 2-h intervals after the 0500 h feeding at 0700, 0900, 1000, and 1300 h (Whitehouse et al., 2024). Blood plasma was separated by centrifugation (1,200 × *g* for 20 min at 5°C). A 4-mL aliquot of plasma was mixed with 1 mL of 15% sulfosalicylic acid, centrifuged again under the same conditions, and the deproteinized plasma was stored at −80°C until plasma AA analysis.

Cows were milked twice daily at 0430 and 1530 h. Milk samples from morning and afternoon milkings were preserved with 2-bromo-2-nitropropane-1,3-diol and composited according to milk yield for analysis of fat, protein, TS, SCC, and MUN (Dairy One Milk Laboratory, Ithaca, NY). Because experimental periods were short (7 d), performance variables (DMI, milk yield, ECM, and milk composition) were considered secondary outcomes in this study and were included primarily to verify that treatments did not negatively affect animal performance during the dose-response evaluation.

Relative bioavailability (**RBV**) of L-Met60 was calculated using slope-ratio methodology. Slopes of plasma responses from infused treatments were used as the reference standard according to previously published approaches (Whitehouse et al., 2017; Rulquin and Kowalczyk, 2003). Relative bioavailability was calculated as

$$RBV,\;\%=\left(\;\frac{slope\;of\;fed\;L-Met60}{slope\;of\;infused\;L-Met60}\;\right)\times 100.$$

Slopes of plasma Met versus grams of infused L-Met60 or fed Smartamine M (Adisseo USA, Alpharetta, GA) were used as a reference standard based on previously published data to calculate the RBV. Sample size was based on logistical constraints and previous dose-response studies evaluating plasma AA responses.

The RSTUDENT Procedure of SAS 9.2 (2010, SAS Institute Inc., Cary, NC) was used to determine outliers (an observation greater than 2.0 SD from the mean) within level and cow for the variables Met (µ*M*), total sulfur AA (**TSAA**; µ*M*), DMI, ECM, and milk protein percent. Outliers were reviewed and removed if justified. A total of 6 observations out of 150 observations were identified as outliers and all data for that observation were removed from both the milk and plasma datasets, resulting in missing values. These missing observations were not imputed; instead, the statistical analysis accounted for them through the use of PROC MIXED, which accommodates unbalanced data structures. Removal of these observations did not alter the overall statistical conclusions.

Plasma Met and TSAA concentrations, as well as milk yield and milk protein concentrations and yields, were analyzed using the PROC MIXED procedure of SAS 9.4 (SAS Institute Inc.) with repeated measures over day. An AR(1) covariance structure was used to model within-cow correlations across sampling days, according to the following model:

*Y_ijklm_* = *μ* + *L_i_* + *P_j_* + *B_k_* + *D_l_* + *LD_il_* + *KC_ilm_* + *E_ijklm_*,

where *Y_ijklm_* = the dependent variable; *μ* = overall mean; *L_i_* = fixed effect of the *i*th treatment (*i* = 1 . . . 5); *P_j_* = fixed effect of the *j*th period (*j* = 1 . . . 5); *B_k_* = fixed effect of the *k*th cow (*k* = 1 . . . 2); *D_l_* = fixed effect of the *l*th day (*l* = 1 . . . 3); *LD_il_* = fixed effect of the interaction between the *i*th treatment and the *l*th day; *K* = regression coefficient of the covariate *C*; *C_ilm_* = value of the covariate variable for the *m*th cow within the *l*th day of the *i*th treatment (*l* = 1 . . . 5); and *E_ijklm_* = random residual ∼*N* (0,). In this model, the random effect of cow(block) was used as the error term for the effect of day and day × treatment interaction. Degrees of freedom were calculated using the Kenward-Roger option of MIXED procedure (SAS, 2010). Significance was noted at *P* ≤ 0.05. The day and day × treatment interaction were not significant; therefore, the means for plasma AA concentration and milk parameters for the 3 d were averaged and this interaction was excluded from the model. The data were weighed for any missing values. The random effect of cow(block) was used as the error term for the effect of treatment level. Least squares means were determined for treatment and treatment means were separated. Level effects were considered as significant at *P* ≤ 0.05 and as trends when *P* > 0.05 to *P* < 0.10. Least squares means were generated and compared, and slope-ratio assay (Finney, 1978) was conducted using PROC REG procedure (SAS, 2010) to generate the linear regression and its coefficient of determination (R^2^).

The NRC (2001) and AMTS were used to evaluate nutrient supply and validate the formulation of the basal diet. According to NRC predictions, the diet supplied 40.6 Mcal/d of NEL compared with a requirement of 42.6 Mcal/d, resulting in a NEL balance of −2.0 Mcal/d. Similarly, AMTS estimated ME supply at 66.6 Mcal/d compared with a requirement of 66.5 Mcal/d, indicating a near-zero ME balance. In both models, milk production supported by energy supply (NEL allowable milk = 38.3 kg/d for NRC; ME allowable milk = 40.9 kg/d for AMTS) was similar to or greater than that supported by MP supply (MP allowable milk = 37.7 kg/d for NRC and 40.9 kg/d for AMTS), indicating that energy supply was not the primary limiting factor for milk production.

Both models predicted adequate lysine supply but a deficiency in Met. Such Met deficiency is common in TMR diets based on alfalfa haylage, heated soybeans, and limited amounts of animal protein sources (Armentano et al., 1997). According to NRC predictions, Met supply expressed as a percentage of MP (Met % MP) and as daily supply (g/d) was approximately 27% lower than recommended values. The NRC model also indicated a deficiency in RDP, which, together with the relatively low MUN values observed in this study, suggests potential urea recycling. Similarly, AMTS predicted that Met supply was approximately 15% below recommended levels. In addition, the Met:ME ratio of the basal diet (0.89 g/Mcal ME) was lower than the recommended range of 1.2 to 1.3 g/Mcal ME (LaPierre et al., 2019; Higgs et al., 2023), further supporting that the basal diet was primarily limited by methionine rather than energy supply.

The 36IL treatment resulted in a 0.06 percentage unit increase in milk protein compared with the control. Other than that, no effect was found among treatments for DMI, milk yield, milk composition, MUN, linear SCS, and milk yield efficiency. Thus, the absence of effect on the performance of dairy cows was similar to fed and infused L-Met groups. This result highlights the opportunity of new experiments with a longer experimental period, focused on milk production and other performance parameters evaluating the dose of 16 g/d to balanced protein supply in the basal diet.

The L-Met60 supplementation did not affect (*P* > 0.05) plasma metabolites (1-methylhistidine, 3-methylhistidine, α-amino-adipic acid, α-amino-butyric acid, β-alanine, carnosine, phosphoserine, and sarcosine), nor did it alter the concentrations of most AA. However, cows receiving either infused L-Met or fed L-Met at the highest level showed significant increases in plasma concentrations of Met (*P* < 0.0001), taurine (*P* = 0.002), and TSAA (*P* < 0.0001) compared with the control group. These increases likely reflect the greater supply of Met available for the methionine–cysteine–taurine pathway, as known as transsulfuration. After intestinal absorption, Met is primarily metabolized in the liver where it can undergo transsulfuration to form cysteine, which serves as a precursor for taurine synthesis through the sequential action of cysteine dioxygenase and cysteine sulfinic acid decarboxylase (Stipanuk, 2004). Consequently, an increased supply of sulfur AA can stimulate this pathway and increase taurine production. The higher plasma taurine concentrations observed in the present study are therefore consistent with increased metabolic flux through the transsulfuration pathway resulting from greater Met availability.

Furthermore, the results of this study support the interpretation that the activation of the transsulfuration pathway is both route- and dose-dependent, since higher levels of infused L-Met resulted in greater plasma concentrations of taurine compared with fed supplement. Likewise, higher levels of fed L-Met resulted in greater plasma concentrations of this AA. This route- and dose-dependent response has been consistently reported in previous studies using the plasma AA dose-response technique (Rulquin and Kowalczyk, 2003; Whitehouse et al., 2017; Ardalan et al., 2021). The regression analysis demonstrated a steeper slope for the infused (0.05424 ± 0.003) compared with fed (0.02354 ± 0.002) supplementation, indicating stronger plasma TSAA response per gram of Met supplied. Consequently, the difference in TSAA among the treatments widened progressively as L-Met60 dose increased, up to 36 g/d.

As shown in Table 2, plasma glutamine concentrations decreased (*P* = 0.004) following Met supplementation. This response may be explained by 2 potential mechanisms. First, elevated supply of Met enhances the antioxidant status in dairy cows (Khan et al., 2023) by promoting synthesis of antioxidant molecules such as taurine and glutathione. This stimulates the transsulfuration pathway and increases the demand of nitrogen donors (Preynat et al., 2009), with glutamine serving as a primary source. Second, increased plasma Met concentrations may alter AA transporter dynamics, potentially reducing glutamine efflux from tissues (muscle and intestine) into the bloodstream due to competition for shared transporters with other AA (Duan et al., 2017). As the current literature lacks consistent reports on glutamine response to L-Met supplementation, further research is needed to clarify the underlying mechanisms of this observation.

**Table 2:** Effects of increasing levels of infused or fed rumen-protected methionine^1^ on lactational performance and plasma AA concentrations in multiparous lactating Holstein cows

Item	Control	18IL	36IL	18FL	36FL	SEM	*P*-value
Performance							
DMI, kg/d	24.2	25.2	24.7	25.2	25	0.84	0.13
Milk yield, kg/d	40.9	40.9	40.7	41.6	40.4	1.52	0.36
Milk fat, %	4.22	4.12	4.16	4.18	4.13	0.1	0.51
Milk protein, %	3.08^b^	3.12^ab^	3.14^a^	3.10^ab^	3.11^ab^	0.04	0.03
Protein yield, kg/d	1.26	1.27	1.28	1.28	1.26	0.04	0.40
Milk lactose, %	4.86	4.9	4.88	4.91	4.85	0.06	0.14
MUN, mg/dL	10.4	10.2	10.5	10.1	10.1	0.47	0.71
ECM, kg/d	45.1	44.6	44.6	45.6	44.1	1.15	0.29
Plasma AA, μ*M*							
Methionine	25.0^c^	32.9^b^	38.3^a^	24.5^c^	24.8^c^	1.86	<0.0001
Lysine	95.3	101.7	96.5	92.1	89.3	6.23	0.15
Histidine	52.6	50.9	52.3	50.2	52.7	3.32	0.86
Cystine	24.2^b^	26.0^a^	26.0^a^	24.3^b^	24.9^ab^	0.83	0.02
Cystathionine and allocystathionine	9.6^c^	13.0^b^	15.8^a^	10.2^c^	10.5^c^	0.64	<0.0001
Taurine	57.0^c^	71.1^ab^	78.1^a^	61.9^bc^	66.7^ab^	4.76	0.002
Glutamine	194.3^a^	153.0^b^	154.5^b^	137.5^b^	143.5^b^	11.06	0.004
TSAA	120^c^	147^b^	162^a^	125^c^	131^c^	6.3	<0.0001

a–c Means in the same row differ (*P* < 0.05).

1 18IL and 36IL mean that 18 or 36 g of L-Met was infused into the abomasum. 18FL and 36FL mean that 18 or 36 g of L-Met from the RP-Met was fed to cows.

The response of circulating sulfur AA to L-Met60 supplementation was dependent on both route of administration and dose. Abomasal infusion resulted in clear increases in plasma methionine and TSAA, whereas oral supplementation did not significantly alter plasma methionine concentrations, despite modest numerical increases in TSAA. This distinction is critical because abomasal infusion bypasses ruminal degradation and represents the metabolizable fraction of methionine, whereas oral supplementation is subject to ruminal losses and variable intestinal availability. The absence of a significant increase in plasma methionine with fed L-Met60 indicates that the metabolizable supply of methionine from the product under practical feeding conditions was limited relative to the infused reference. Accordingly, increases in plasma methionine were observed only under abomasal infusion conditions.

Despite this, TSAA concentrations increased numerically with oral supplementation and exhibited a dose-related trend, although less pronounced than with infusion (Figure 1). This suggests that a portion of the supplemented methionine was absorbed but rapidly used through metabolic pathways such as transsulfuration, thereby limiting accumulation of free methionine in plasma. Increases in taurine and cystathionine support enhanced sulfur AA metabolism, although flux through this pathway was not directly measured.

**Figure 1:**
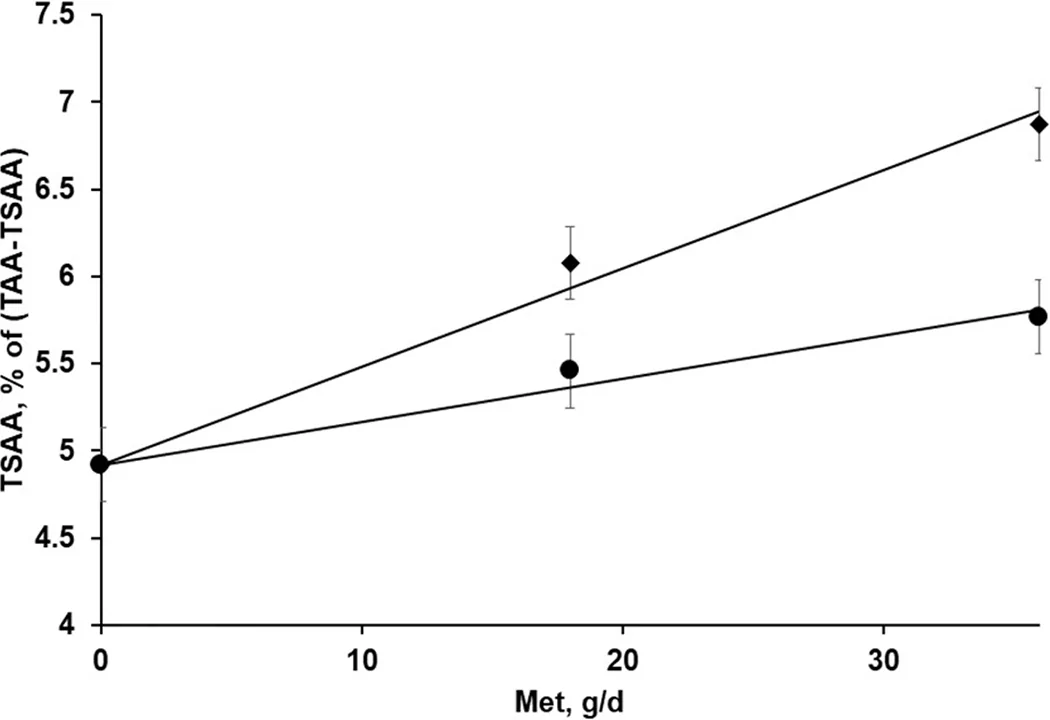
Relationship between methionine supply (infused or fed) and plasma total sulfur amino acid (TSAA) concentrations used to estimate relative bioavailability of rumen-protected methionine in multiparous lactating Holstein cows. Infusion (♦): Y = 4.98 + 0.05424; slope SE = 0.003, intercept SE = 0.05, R^2^ = 0.99. LipoAktiv L-Met60 (•): Y = 4.98 + 0.02354; slope SE = 0.002, intercept SE = 0.05, R^2^ = 0.96; relative bioavailability = (0.02354 ÷ 0.05424) × 100 = 43.4 ± 2.5. Error bars indicate SEM. TAA = total amino acids.

Although previous studies have used plasma methionine as the primary response variable, TSAA was used in the present study to capture the integrated metabolism of sulfur AA, including downstream conversion products. Plasma methionine and TSAA responses were strongly associated, supporting the use of TSAA as an indicator of metabolizable methionine supply under conditions of rapid metabolic utilization.

Relative bioavailability was estimated using slope-ratio methodology based on plasma TSAA responses. Importantly, RBV reflects the relative efficiency of conversion of supplemented methionine into metabolizable supply compared with the infused reference, rather than absolute increases in plasma methionine concentrations. The estimated RBV of 43.4% indicates that L-Met60 provides a measurable but limited source of metabolizable methionine under the conditions evaluated.

These results highlight the importance of distinguishing between infusion-based experimental models and practical feeding scenarios. Whereas abomasal infusion provides a controlled reference for determining bioavailability, oral supplementation outcomes are influenced by ruminal degradation and postabsorptive metabolism. These findings suggest that optimization of product protection or inclusion rate may be required to achieve detectable plasma responses under practical feeding conditions.
